# When is it best to test? Attitudes of health professionals regarding genetic testing for Familial Adenomatous Polyposis (FAP)

**DOI:** 10.1186/1897-4287-10-S2-A43

**Published:** 2012-04-12

**Authors:** E Lynch, RE Duncan, F Macrae, M Delatycki

**Affiliations:** 1 Austin Health Clinical Genetics Service, Australia; 2 Bruce Lefroy Centre for Genetic Health Research, Murdoch Childrens Research Institute, Australia; 3 Centre for Adolescent Health, Children’s Bioethics Centre, Murdoch Childrens Research Institute, Australia; 4 Department of Colorectal Medicine and Genetics, The Royal Melbourne Hospital, Australia

## 

Familial Adenomatous Polyposis (FAP) is a well described autosomal dominant syndrome, whereby individuals develop multiple (up to thousands) of adenomatous polyps in the large bowel, conferring an extremely high risk of bowel cancer if left untreated.

If the family specific mutation is known, genetic testing can be offered to at risk individuals to determine the need for endoscopic surveillance. Guidelines suggest starting endoscopic surveillance from the early teens. Most individuals with FAP undergo colectomy between the ages of 15 and 25 years [1].

Recent studies have shown that many adults with FAP want their children to undergo genetic testing before the age of 12 years, the approximate age at which genetic testing is usually offered in Australia [2,3].

Health professionals working in cancer genetics in Australia and New Zealand completed a web based survey aimed at examining their attitudes and experiences regarding genetic testing for young people at risk of FAP.

Findings from the survey provide an insight into the views and practices of health professionals working in this area, including: the age at which they believe testing young people is most appropriate; their experiences regarding parental requests for testing of younger children; and whether the option of prenatal testing and pre-implantation genetic diagnosis is routinely discussed with individuals who have FAP and are also of child bearing age. These have important implications for clinical practice within the Australian cancer genetics community.
